# One-Stage Urethroplasty for Strictures in Maiduguri, North Eastern Nigeria

**DOI:** 10.5402/2012/847870

**Published:** 2012-03-04

**Authors:** Ahmed Gadam Ibrahim, Nuhu Ali, Sulieman Aliyu, Abubakar Alhaji Bakari

**Affiliations:** Department of Surgery, College of Medical Sciences, University of Maiduguri and University of Maiduguri Teaching Hospital, P.M.B., Borno State, Maiduguri 1414, Nigeria

## Abstract

*Background*. Urethral stricture is a frequent cause of lower urinary tract obstruction worldwide. The aim of this study is to present our experience with one-stage urethroplasty. *Methods*. All males that underwent one-stage urethroplasty between January 2001 and December 2010 were retrospectively reviewed. Details of their biodata, clinical presentation, diagnostic investigations, operative treatment, postoperative complications, and other outcome of surgery were extracted and analyzed. *Results*. Ninety-one patients aged 8–76 years, (mean; 45.6 ± 19.7) with urethral stricture were studied. Postinfective strictures accounted for 58.2% and postprostatectomy strictures for 3.3%. Twenty-six (27.9%) of the strictures were in the posterior urethra of which 18 (59.2%) were posttraumatic. Fifty-seven strictures (61.3%) were in the anterior urethra of which 51 (54.8%) were postinfective. Thirty-nine (42.9%) patients had end to end anastomosis, 29 (31.9%) flap augmentation and 17 (18.7%) tabularized flap substitution, and 6 (6.6%) dorsal onlay grafts (5 with buccal mucosa and 1 with penile skin). There were 18 (19.8%) cases of wound infection, 12 (13.2%) of restricture and 6 (6.6%) cases of urethrocutaneous fistula. Satisfactory urinary stream was found in 77 (84.6%) patients. There was no mortality. *Conclusion*. Infection is the commonest cause of urethral stricture followed by trauma, and one-stage urethroplasty give excellent results.

## 1. Introduction

A urethral stricture is caused by narrowing of the urethral lumen due to spongiofibrosis, resulting in loss of distensibility and compliance, leading to poor urinary stream which may lead to further complications. It is a common problem worldwide affecting mainly the male urethra [1]. The aetiology of acquired urethral strictures varies from inflammatory causes to traumatic scarring after blunt perineal/pelvic trauma and iatrogenic causes following surgery or urethral catheter use [2, 3].

Most postinfective strictures are located in the anterior urethra (bulbopenile), whereas posttraumatic strictures affect the bulb or cause posterior urethral disruption or distraction the latter is a serious challenge to the urologist, because they are often associated with significant complications including incontinence and erectile dysfunction [4].

Various forms of repair of the urethra have been developed and perfected over the years, ranging from excision and end-to-end anastomosis in short segment strictures to substitution urethroplasty in long segment strictures. The aim is to produce a wider, stable, and more compliant or distensible urethra that will produce a positive impact on voiding [5, 6].

We undertook one-stage approach because, in the interval between the first and second stages, infections of the operating site are common, making the second stage of the operation more difficult; furthermore, most of our patients are of the low socioeconomic status and can afford surgery only once in their life time. We managed 91 patients by one-stage urethroplasty at the UMTH over a 10-year period; this retrospective study details the outcome of our intervention.

## 2. Patients and Methods

All males that underwent one-stage urethroplasty for urethral stricture at the UMTH between January 2001 and December 2010 were retrospectively reviewed. Details of their biodata, clinical presentation, diagnostic investigations, operative treatment, postoperative complications, and other outcome of surgery were extracted from their hospital records and analyzed. The diagnosis of urethral stricture was made on clinical assessment, a retrograde urethrogram (RUG) and micturating cystourethrogram (MCUG). The patients had initial urinary diversion by a suprapubic cystostomy (SPC). One-stage urethroplasty was performed on all patients after resuscitation where necessary, ensuring optimal renal function and treating any urinary tract infection with antibiotics based on sensitivity. Penile skin flap, substitution, or augmentation urethroplasty were the main surgical techniques used. With a few buccal mucosal on-lay grafts, short segment strictures, and those from posterior urethral disruption, had end-to-end anastomosis after excision of the stricture, while long segment or beaded postinflammatory strictures were repaired by penile skin flaps. Calculi, bladder diverticuli, abdominal wall hernias were treated at the same surgery or after the urethroplasty have healed. The postoperative outcome was carefully documented. Thirteen patients with incomplete medical records were excluded from the study. 

## 3. Results

A total of 91 patients had one-stage urethroplasty during the study period. Their mean age was 45.6 ± 19.67 years (range 8–76). The 41–50-year-age group was the most affected accounting for 20 (21.9%) patients (Table 1).The causes of urethral stricture are outlined in Table 2 which included post-infective in 53 (58.2%), post-traumatic in 30 (32.9%), iatrogenic, as a complication of urethral catheterization in 5 (5.5%) and as a complications of prostatectomy in 3 (3.3%) patients. Sixty-seven (72%) strictures were in the anterior urethra comprising 26 (38.8%) bulbar, 34 (50.7%) bulbo-penile, and 7 (10.4%) penile. Twenty-six (28.0%) of the strictures were in the posterior urethra Table 3. Most of the patients presented with progressive poor urinary stream that improved with straining. Eleven (12.1%) presented in acute urinary retention, while 6 (6.6%), presented with multiple perineal urinary fistulae (watering can perineum). The mean duration of symptoms was 24 months for the postinflammatory strictures, and 3–6 months for the posttraumatic strictures.

Resection of the strictures and primary anastomosis were done in 39 (42.9%) patients while substitution urethroplasty (augmentation and replacement with penile or scrotal skin or buccal mucosa) was performed in 48 (52.7%) patients (Table 4). All the patients had sterile urine on culture before the procedure. General anaesthesia was used in 9 (9.9%) patients 5 of which had buccal mucosal graft. The rest were done with spinal anesthesia. The mean duration of surgery was 98.25 ± 17.8 minutes (range 60–120) for resection and en-to-end anastomosis, while substitution urethroplasty took a little longer, with a mean operation time of 125.4 ± 23.4 minutes.

The postoperative complications (Table 5) included urethral fistula in 6 (6.6%) patients, out of which 4 (66.7%) had flap augmentation and 2 had tabularized pedicled penile skin flap. None of the resection and end-to-end anastomosis patients had fistula formation. Wound infection was seen in 18 (19.8%) patients, made up of 15 (83.3%) that had substitution urethroplasty (augmentation 9, and replacement 6) and 3 (16.7%) that had resection and anastomosis. Restricture was seen in 12 (13.2%) patients, of which 7 (58.3%) had resection and anastomosis and 5 (41.7%) had substitution urethroplasty. Four fistulae healed spontaneously on suprapubic cystostomy drainage, while 2 were repaired. Majority of the patients, 67 (73.6%), did not have any postoperative complications.

Seventy-seven (84.6%) of the patients had satisfactory voiding at discharge with a urinary flow rate of at least 10–15 mls/sec, and eleven (12.1%) patients had maximal flow rate of over 15 mls/sec on discharge. The mean follow-up duration was 3 months, and majority of the patients were lost to followup after 6 months.

## 4. Discussion

Urethral stricture disease has been a common urological problem in the West African subregion from time immemorial. The vast majority of urethral strictures in this part of the world present late with many complications, and this may be because of the social stigma associated with genital problems. Urethral stricture disease affects all age groups but is most common in males in their prime of life with mean age ranging between 30 and 50 years [5–7].

In the past, post-infective urethral strictures especially following gonococcal urethritis predominated [7]; however, there is now a changing pattern of aetiology with trauma accounting for most cases [8, 9]. In this study, postinfective strictures were still more common in variance with what is obtainable in other parts of Nigeria [10]. This may be due to higher urbanization in those parts of the country leading to more automobile accidents and other forms of trauma. Other causes of urethral stricture of particular interest include posturethral catheterization and postprostatectomy [11, 12]. In this study there were 3 cases of urethral strictures following traumatic catheterization and another 3 following prostatectomy. One of the postprostatectomy strictures in this series were managed by serial dilatation, anastomotic urethroplasty and flap augmentation. This is in contrast to Onuigbo et al*'*s [11] experience where all their patients were managed by serial dilatation with good results. The choice of modality of treatment depends on extent of fibrosis, site, and length of the stricture.

 In the management of anterior urethral strictures, resection with spatulation, followed by mucosa to mucosa tension-free end to end anastomosis of the urethra, was the most frequently performed procedure in our series because most of the strictures were short segment, solitary, and located in the anterior urethra. Such alignment of the urethra is responsible for the high success rate of anastomotic urethroplasty, which is in excess of 90% and sustained on long-term basis [13, 14]. Patients with complicated strictures for example, urethro-cutaneous fistulae, extensive periurethral fibrosis, false passages from previous attempts at dilatation, diverticuli, and others, require more robust surgical technique which entails maximal excision of scar tissue with conservation of the ischiocavernosus and bulbospongiosus muscles responsible for ejaculation at the same time dissecting in the mid-line to protect the nervi erigentis proximally particularly at the bulbomembranous urethra. This group of patients requires substitution urethroplasty by way of augmentation or total replacement of the diseased segment. The success rate of these surgeries is measured by increase in urine flow rate and patients satisfaction. The success rate has been variously reported to range between 40 to 80%. In this study 77 (84.6%) had satisfactory voiding as indicated by urine flow rate of 10–15 mL/s; however, it was difficult to assess the long-term impact of substitution urethroplasty on voiding because of poor followup. Most of the patients were lost to followup by the first 6th postoperative month.

The postoperative complications are similar to earlier reports, with wound infection being the most common. Restricture is common in patients that had resection, and end-to-end anastomosis and fistulae are mostly seen after substitution urethroplasty with most closing spontaneously.

## 5. Conclusion

Urethral stricture disease is a common problem in north-eastern Nigeria with the infective causes still predominating in some areas although traumatic causes are on the rise. One-stage urethroplasty is most appropriate with acceptable outcome in our environment considering our prevailing social and economic factors.

## Figures and Tables

**Table 1:** Age distribution of patients.

Age (years)	Frequency	Percentage
<10	2	2.2
11–20	4	4.4
21–30	18	19.8
31–40	17	18.7
41–50	20	21.9
51–60	13	14.3
61–70	13	14.3
71–80	4	4.4

Total	91	100

**Table 2:** Aetiology of strictures in 91 patients.

Aetiology	Frequency	Percentage
Postinfective	53	58.2
Posttraumatic	30	32.9
Iatrogenic	5	5.5
Postprostatectomy	3	3.3

Total	91	100

**Table 3:** Site and aetiology of urethral stricture in 91 patients.

Aetiology	Site			
	Posterior	Bulbar	Bulbo-penile	Penile
Postinfective	—	17	29	5
Posttraumatic	18	7	4	1
Iatrogenic	5	2	1	1
Postprostatectomy	3	—	—	—

Total	26	26	34	7

**Table 4:** Operative procedures.

Type of urethroplasty	Penile (skin)	Scrotal (skin)	Others	Total (%)
Resection and anastomosis	—	—	—	39 (42.9)
Augmentation				
Flap	23	6	—	29 (31.9)
Graft	1	—	5	6 (6.6)
Substitution				
Flap	16	1	—	17 (18.7)

Total	40	7	5	91 (100)

Key: others: buccal mucosal graft.

**Table 5:** Postoperative complications.

Complications	Resection and anastomosis (%)	Augmentation (%)	Substitution (%)
Wound infection	3	9	6
Restricture	7	4	3
Fistula	—		2
Total	10	13	11
